# Dietary fat and antioxidant vitamin intake in patients of neurodegenerative disease in a rural region of Jalisco, Mexico

**DOI:** 10.1179/1476830513Y.0000000089

**Published:** 2014-11

**Authors:** Mónica Navarro-Meza, Genaro Gabriel-Ortiz, Fermín P. Pacheco-Moisés, José A. Cruz-Ramos, Antonio López-Espinoza

**Affiliations:** 1 Departamento de Salud y Bienestar, Laboratorio de Biología Molecular e Inmunología, Centro Universitario del Sur. Universidad de Guadalajara, Ciudad Guzmán, Jalisco, México; 2 División de Neurosciencias, Centro de Investigación Biomédica de Occidente del Instituto Mexicano del Seguro Social, Guadalajara, Jalisco, Mexico; 3 Departamento de Química, Centro Universitario de Ciencias Exactas e Ingenierias, Universidad de Guadalajara, Guadalajara, Jalisco, México; 4 Departamento de Cultura, Arte y Desarrollo Humano, Centro de Investigaciones en Comportamiento Alimentario y Nutrición, Centro Universitario del Sur. Universidad de Guadalajara, Ciudad Guzmán, Jalisco, México

**Keywords:** Intake of antioxidant vitamins, Lipids, Neurodegenerative diseases, Rural region

## Abstract

**Objective:**

To evaluate and compare the intake of lipids and (A, E, and C) vitamins in patients with and without possible neurodegenerative diseases.

**Methods:**

Twenty adults with possible Alzheimer's disease or Parkinson's disease and 41 control subjects (50–89 years old) from a rural region were studied. Dietary intake was evaluated with the analysis of macronutrients and micronutrients conducted by a food frequency questionnaire and 24 hours dietary record. Analyses were adjusted for age, sex, body mass index, and energy intake. Through interrogation and use of medical record form of health secretary we obtained information about the sociodemographic characteristics. Multivariate analysis of variance to allow for covariated adjustment was used.

**Results:**

Patients had a lower energy intake, vitamin C (*P* = 0.016), fruits (*P* < 0.001), vegetables (*P* = 0.037), and oils and fat (*P* = 0.002), than the controls. Interestingly, the C vitamin intake in patients was still higher than the recommended. Patients had a higher consumption of cereals (*P* = 0.017), high-animal fat diet (*P* = 0.024), and whole milk (*P* < 0.001); 2.4% of the controls smoke and 5% are alcohol consumers. Eighty-five percent of patients and 78% of the controls do not have physical activity. Family history of subjects in this study indicated chronic diseases.

**Conclusion:**

The subjects included in this study had a high intake of C vitamin, this is due to the consumption of fruits and vegetables. However, patients with possible Alzheimer's or Parkinson's disease had a lower intake of fruits and vegetables, which could be due to type of food to which they have access.

## Introduction

Neurodegenerative diseases are a complex and multifactorial disorder that affects the central nervous system (CNS), such as Alzheimer's (AD) and Parkinson's disease (PD). These provoke the loss and death of neuronal connections, as well as oxidative stress responses.^1,2^ Inadequate intake of fat and antioxidant vitamins has been associated with AD and PD.^3–6^ It has been suggested that consumption of antioxidant vitamins such as: A, C, and E, could present protective effects on the development of these diseases.^7–9^ It has been reported that an inadequate intake of vitamins is due to a low consumption of fruits and vegetables, a high intake of saturated fat, especially animal, and also an inappropriate lifestyle, mainly due to physical inactivity and sedentary lifestyles.^6,8,10,11^

In the experimental models of AD, it has been reported that diets rich in docosahexaenoic acid have antioxidant properties and improve cognitive impairment.^12^ On the other hand, it has been suggested that the consumption of saturated fatty acids and cholesterol is associated with cognitive impairment and dementia, monounsaturated fatty acids (MUFAs) have protective effects while polyunsaturated fatty acids (PUFA) may influence the risk of cardiovascular disease.^13^ It has been suggested that concentrations of MUFA and PUFA acids could be related to the antioxidant properties of vitamins, which suggests that adequate levels of these vitamins may occur during the process of regeneration of tocopherol, since vitamin C may potentiate antioxidant activity.^14^ An important source of vitamins is fruits and vegetables; these food groups have beneficial effects on health and body mass index (BMI). According to National Survey of Health and Nutrition (ENSANUT), 2006 in Mexico, the average consumption of fruits and vegetables is 116.3 and 122.6 g/day, a lower value than the recommended.^15^ Little information is available about the representative food consumption in rural areas. Economic, environmental, cultural, and demographic factors, have been identified as decisive in the quality of a diet, this is why it is important to evaluate the intake of macro and micronutrients in the Mexican population.^16,17^ The limited epidemiological evidence available on fruit and vegetable consumption and cognition generally supports a protective role of these macronutrients against cognitive decline, dementia, and AD. The dietary antioxidant intake in foods is superior to supplements in human studies on cognition and risk of developing AD.^18,19^

The aim of this work is to evaluate and compare the dietary intake (antioxidant vitamins, and macronutrients) in adult patients with neurodegeneration attended at two rural health centers (Ciudad Guzman and Teocuitatlan de Corona, Jalisco, Mexico), and their respective controls, without neurodegeneration. The age range of the sample was 50–87 years.

## Subjects and methods

A cross-sectional, descriptive, and comparative study was conducted with 20 patients diagnosed with neurodegeneration (AD and PD) and 41 elderly controls (without neurodegeneration), ranged from 50 to 89 years old. Subjects were recruited from the Ministry of Health Services, from the Health Centers at Ciudad Guzman, and Teocuitatlan de Corona, Jalisco, from January to December 2012. The inclusion criteria was: patients with neurodegeneration according to the International Statistical Classification of Diseases and Health Related Problems (ICD-10), which was conducted by specialists, certified Health Sectors, and 50-year-old seniors who attended the services. The exclusion criteria was: adults that are not diagnosed with neurodegeneration. Patients who were administered drugs or drug-related Parkinsonism induction, patients with a stroke or accident, and patients with early cognitive impairment were not included. The control group included 41 subjects matched by sex, age, and socioeconomic status, who attended service at the health centers and without alterations of their nutritional status. This protocol was approved by the Institutional Research and Bioethics Committee (México). A medical doctor specialist with experience in movement disorders determined the Hoehn and Yahr score, and on the score Unified Parkinson's Disease Rating Scale (UPDRS) notes total activities of daily living (ADL), in both the on and the off phase.

## The population study

The study area consists of Guzman City, Jalisco, and the municipality of Teocuitatlan de Corona, Jalisco. The population of Guzman City is 100 534 habitants (The Institute National of Statistics and Geography) (INEGI, 2010)^20^. The Southern Region of Jalisco is formed by 16 townships, (INEGI)^20^ in Guzman City; 1083.4 approximately are adults (more 60 years old) population in Teocuhitatlan and 11% of total population in Guzman City^20^; in the present study included only adults. This local area, instead of the whole province was chosen in order to achieve a complete identification of patients. Patients were gathered from January to December 2012. Information was obtained from the controls and newly diagnosed patients; characteristics of patients and controls are shown in Table 1.

**Table 1:** Selected sociodemographic characteristics of patients with neurodegenerative disease (PD and AD) and controls (mean ± SD)

	Patients (*n* = 20)	Controls (*n* = 41)
Age, years	66 ± 2	72 ± 1
Energy intake (kJ/day)	6787 ± 862	10383.26 ± 816
Body mass index (kg/m^2^)	24.8 ± 1.3	28.7 ± 0.84
Female, number (%)	4 (7%)	34 (57%)
Male, number (%)	16 (26%)	6 (10%)
Education (schooling years), number (%)		
No schooling	7 (35%)	4 (9.7%)
Elementary school	9 (45%)	28 (70.3%)
High school	3 (15%)	6 (15%)
> 12 years	1 (5%)	2 (5%)
Socioeconomic status, number (%)		
Low	12 (60%)	18 (45%)
Medium	6 (30%)	14 (35%)
High	2 (10%)	9 (22.5%)
Occupation, number (%)		
Housewife	8 (40%)	34 (85%)
Rural works	0	6 (15%)
Marital status		
Single	2 (10%)	11 (27%)
Married	15 (75%)	19 (46%)
Widower	3 (15%)	11 (27%)
Inactive, number (%)	17 (85%)	32 (78%)
Physical activity, no (%)	3 (15%)	9 (22%)
Family history, number (%)		
Alzheimer's disease	4 (20%)	2 (4.9%)
Cancer	2 (10%)	1 (2.4%)
Liver cirrhosis	1 (5%)	
Diabetes mellitus	6 (30%)	18 (44%)
Dyslipidemia		3 (7.3%)
Epilepsy	1 (5%)	
Hypertension	6 (30%)	11 (26.7%)
Hypothyroidism		2 (4.8%)
Renal disease (insufficiency)		1 (2.4%)
Current smoker, number (%)	0	1 (2.4%)
Alcohol consumers, number (%)	0	2 (5%)

## Variable analysis

### Determination of anthropometric parameters

The measures of body weight and height were considered in determining the BMI; they were classified according to criteria taken from reports of the World Health Organization; malnutrition (BMI < 18.5), normal (BMI, 18.5–24.9), overweight (BMI, 25.0–29.9), and obese (BMI ≥ 30.0).^21^

### Food consumption analysis

Adults included in this study underwent a survey of food consumption frequency on a daily basis, a 24-hour recall^22,23^ was used which included 186 foods, or 15 different food groups. The obtained dietary intake data were converted into average grams (g), milligrams (mg), or micrograms (µg) of food consumed per person a day. Interviews were conducted by a certified nutrition professional and the daily average intake of kilocalories (kcal) consumed according to macronutrients was determined. Subsequently, the percentage and grams (g) of macro and micronutrients were determined.^21,24^ Each patient was applied three reminders for 24 hours and frequency of food consumption based on the following: in consumption frequency were added to some foods such as pitahaya, Mexican snacks, and some alcoholic drinks commonly found in the region. The consumption frequency was conducted with the support of images representing different foods and portions (cups, plates, and spoons). The results were transferred to the database, and food information processing support was used for the composition of food and the Mexican equivalent table; the energy supply, consumption macronutrients, A, E, and C vitamins, and folic acid were calculated with the help of food composition and recommendations.^25,26^ The adequacy percentage was calculated by using the recommendations of energy consumption, macronutrients, and micronutrients for adults; we supported in.^16^ The adequacy was calculated for energy and selected macro- and micronutrients (protein, fat, carbohydrates, vitamin A, vitamin E, vitamin C, and folate). Percent adequacy compares the estimated average daily nutrient intake relative to the dietary reference intakes^27,28^ for energy and each analyzed nutrient based on the age and sex of each individual; the estimated average was compared daily and nutrient intake is expressed in grams, milligrams, or micrograms for micronutrients or macronutrients. Incomplete data related to the food survey were excluded.

### Statistical analysis

The data were tabulated and analyzed statistically with SPSS version 19.0. It was calculated with a descriptive statistics and frequency distribution. The results are presented as means and standard deviations (SDs). Adjustments were made for age, sex, BMI, and kilocalories, the significance of differences between means was calculated by multivariate analysis. A *P* value <0.05 was considered significant.

### Ethical considerations

All of the participants signed an informed consent form prior to the survey interview. The survey and written informed consent forms were approved by the Ethics Committee of the Institute of Public Health, VI region (México).

## Results

### Baseline characteristics of patients and adults

Participants ranged in age from 50 to 89 years, 20 subjects (33%) presented a diagnosis of neurodegeneration (PD and AD) with an average age of 68 ± 2 years and 41 (67%) were female. Thirteen PD patients (12 men and 1 woman) and AD patients (3 women and 4 men). Fifty-six (56%) had moderate-to-severe disease (Hoehn and Yahr stages 2.5–5). Mean ADL UPDRS score was 21 ± 14 (range, 0–52). Mean UPDRS motor score in on phase was 15.22 ± 8 (range, 0–72). Ninety-two (92%) of PD patients were on levodopa therapy and eight (8%) were taking a dopamine agonist. All of the patients treated with levodopa had some motor fluctuations. The clinical dementia rating scale was applied in AD patients: 66% (2 stage) and 33% (0.5 stage).

In the group with neurodegeneration, 80% (*n* = 16) were men and 20% (*n* = 4) were women; 60% showed a low socioeconomic level, 30% had a middle-class level, and 10% a higher socioeconomic level. 35% of the patients reported not having attended school, 45% only primary, 15% attended secondary, and 5% reported having schooling for more than 12 years. 40% of the patients reported engaging in housework, 85% have physical inactivity, and 75% are married. Family history of the subjects in this study indicated diabetes mellitus (30%), hypertension (30%), and AD (20%) (Table 1).

Kilojoules consumed per day in the patients group were 6787 ± 862 and BMI was 24.8 ± 4 kg/m^2^, corresponding to normal weight. Macronutrient consumption between groups did not show statistically significant differences. Protein intake was greater (*P* = 0.293), lower lipids intake (*P* = 0.688), and carbohydrate (*P* = 0.653) compared with the control group (Table 2). With regard to micronutrient consumption, vitamin C intake was lower in patients compared with the control group (*P* = 0.016). No statistically significant differences between groups were found for the intake of vitamin A (*P* = 0.542), vitamin E (*P* = 0.689), folic acid (*P* = 0.141), cholesterol (*P* = 0.386), MUFA (*P* = 0.803), and PUFA (*P* = 0.152) (Table 2). Patients showed a higher consumption of cereals (*P* = 0.017), high-animal fat (*P* = 0.024), and whole milk (*P* < 0.001), and a low intake of vegetables (*P* < 0.001), fruits (*P* = 0.037), and oil and fats (*P* = 0.002) (Table 3). 55% of the patients consumed less carbohydrates than the recommended, 100% consumed more proteins than the recommended, 85% less than the recommended fat, 25% less of vitamin C, 75% had insufficiency in the recommended value of vitamin A and 50% of cholesterol, 100% less of the suggested vitamin E, 75% less than the recommended folic acid, 90% reduced the amount of recommended MUFA intake, and 70% underconsumed the recommended PUFA intake (Table 4). The control group shows an average age of 72 ± 1 years, 43% (*n* = 7) were men, and 57% (*n* = 34) were women. 45% presented a low socioeconomic level, 35% a medium level, and 20% had a high level; 9.7% reported not having attended school, 70% only primary, 15% attended secondary, and 2% reported having completed more than 12 years of education. 2.4% are smokers, 5% are alcohol consumers, and 78% of controls exhibit physical inactivity, 46% of them are married. The family history of the subjects in this study indicated diabetes mellitus (41.4%), hypertension (24%), and cerebrovascular events (12%) (Table 1). Kilojoules consumed per day were 10393 ± 815 and 28.7 ± 5 kg/m^2^, which are an overweight indicator (Table 1).

**Table 2:** Macronutrient and micronutrient intake in patients with neurodegenerative disease (PD and AD) and controls (mean ± SD)

Intake	Patients (*n* = 20)	Controls (*n* = 41)	*P*
Protein intake (g)	82 ± 8	68 ± 5	0.293
Fat intake (g)	70 ± 13	74 ± 8	0.688
Carbohydrate intake (g)	328 ± 45	357 ± 28	0.653
C vitamin (mg)	233 ± 71	468 ± 45	0.016*
A vitamin (µg)	970 ± 231	1158 ± 146	0.542
E vitamin (mg)	7 ± 1	7.5 ± 0.6	0.689
Folic acid (µg)	236 ± 17	345 ± 11	0.141
Cholesterol (mg)	306 ± 450	827 ± 284	0.386
MUFA (g)	24 ± 5	25 ± 3	0.803
PUFA (g)	19 ± 2	13 ± 1	0.152

* corresponds to the significance of p ≤ 0.05 Values were adjusted for sex, kilocalories, BMI, and age.

**Table 3:** Intake of food groups in patients with neurodegenerative disease (PD and AD) and controls (g/day)

Food groups	Patients (*n* = 20)	Controls (*n* = 41)	*P*
Vegetables	156 ± 41	405 ± 2	0.000*
Fruits	311 ± 125	665 ± 79	0.037*
Cereals	438 ± 44	293 ± 27	0.017*
Leguminosae	50 ± 26	90 ± 17	0.267
AOA very low in fat	21 ± 6	30 ± 4	0.302
AOA low in fat	22 ± 8	24 ± 5	0.877
AOA moderate in fat	46 ± 10	24 ± 6	0.116
AOA high in fat	14 ± 3	3 ± 2	0.024*
Semi-skimmed milk	67 ± 43	157 ± 27	0.118
Whole milk	228 ± 30	40 ± 19	0.000*
Oil and fats	4 ± 4	22 ± 2	0.002*
Oil and fats with proteins	9 ± 3	14 ± 2	0.285
Sugar without fat	54 ± 26	48 ± 16	0.855
Sugar with fat	5.9 ± 3	3.4 ± 2	0.636
Alcohol	0.92 ± 0.06	0.089 ± 0.040	0.033*

Values are means ± SD, analyses were adjusted for sex, kilocalories, BMI, and age.

AOA, animal foods. * corresponds to the significance of p ≤ 0.05

**Table 4:** Nutrient intake adequacy and the percentage of inadequate intake (minor < to reference values)

	Patients (*n* = 20)			Control (*n* = 41)		
	Median	Adequacy of median (%)	Less reference value patients numbers (%)	Median	Adequacy of median (%)	Less reference value patients numbers (%)
Energy (kJ, %)/day	5597.75 (2788.4–18664.75)	67 (33.4–223)	85	9838.98 (900–20054)	118 (10.7–240)	27
Carbohydrates (g, %)/day	212.75 (41–661)	85.18 (16–264.8)	55	351 (43–937)	140 (17–185)	22
Protein (g, %)/day	85.18 (43–146)	138.9 (103–352.9)	0	61 (7.6–185)	148 (18–447)	20
Fat (g, %)/day	26.37 (6–182.9)	38 (8.8–268.3)	85	57 (1.3–280)	84 (1.9–411)	61
C vitamin (mg, %)/day	116.09 (6–979.18)	177 (8.7–1494.93)	25	461 (329–2252)	704 (507–3464)	2
A vitamin (µg, %)/day	312.85 (11.93–2504)	56 (2.15–451)	75	920 (5.7–4280)	165 (1.02–771)	56
E Vitamin (mg, %)/day	5.7 (1.4–19.2)	28 (7–96)	100	8.1 (6.14–15.25)	40 (30.7–76.25)	100
Folic acid (µg, %)/day	139.24 (24.31–880)	43 (7.5–275)	75	240 (190–1109)	75 (59–346.5)	32
Cholesterol (mg, %)/day	196.5 (34–826)	98 (17–413)	50	93 (32–6430)	46.5 (59–346.5)	58
Monounsaturated fat (g, %)/day	10.8 (0.47–54.18)	43.3 (1.88–216.72)	90	18.41 (9.69–36.4)	73 (53–372)	66
Polyunsaturated fat (g, %)/day	10.95 (3.4–50.34)	60.8 (18.8–279)	70	13.87 (3.4–50.34)	77 (53–202)	71

Reference values: energy intake, 1985 kcal/day, carbohydrates 70%/day, protein 0.8 g/kg/day, fat 30%/day, C vitamin 50 mg/day, A vitamin 1000 µg/day, E vitamin 20 mg/day, folic acid 200 µg/day, cholesterol 200 mg, monounsaturated fat 20%, and polyunsaturated fat 35%. The adequate percentage was calculated for energy and selected macro–micro nutrients, and the estimated average of daily nutrient intake relative to the dietary reference intake for energy and each analyzed nutrient based on age.^27,28^

We observed in the controls a higher intake of vegetables (*P* < 0.001), fruits (*P* = 0.037), cereals (*P* = 0.017), and oils and fats (*P* = 0.002) compared with the patients, Table 3. In general, this study showed an increase in food consumption in the following groups: cereals, foods with fat, sugar, and whole milk (Table 3).

In the control group, 22% consumed less carbohydrates than the recommended, 20% consumed less protein than the recommended, 61% consumed less than the suggested fat, 2% less of the vitamin C, 56% had insufficiency of vitamin A and 32% of folic, 100% less of the suggested vitamin E, 66 and 71% reduced the amount of recommended MUFA and PUFA intake (Table 4).

## Discussion

Our study reports an energy consumption in patients less than 2000 kcal/day, this result is consistent with those that are shown in the ENSANUT, 2006.^29^ This survey indicated that rural adults have an average consumption of 1644 kcal/day, and adults in the states of central México: Jalisco, Michoacán, Nayarit, and Querétaro, have a consumption of 1718 kcal/day, with a prevalence of 28% being of normal weight and 39% being overweight.^16^ While in another study which evaluated food consumption in PD patients and controls,^30^ the evaluation of anthropometric parameters and food intake reported an average of 1866 kcal/day and a BMI indicating overweight in both patients and controls. The results of our study suggest that the decrease in energy intake of the patients is probably due to the development of neurodegeneration itself, while other studies showed no significant differences compared with the control group.^28,29^ However, the average consumption reported by patients is similar to what we found in this study, a reported consumption characteristic might be due to the season in which the survey was recorded or the characteristics of the survey. In addition, nutritional intake may be associated to neurodegeneration. In this regard, it has been reported that olfactory dysfunction in PD patients may alter the nutritional status which in turn may accelerate the development of neurodegeneration.

We found that 30% of patients and 19% of controls have less consumption power at baseline, Table 4.^27,28^ ENSANUT 2006, reported that adults had a lower energy intake in addition to presenting obesity. In this study, it was found a lower energy intake in the neurodegeneration group compared with the control group; BMI normal, corresponding to patients, and in the control group indicating being overweight^31^ (Table 1), suggests that other factors may have an influence, such as socioeconomic status, and the greater proportion of subjects both patients and controls showed a low socioeconomic level (Table 1). This issue that has been reported as a positive association between BMI and rural population^32^ is likely to be related to changes in patterns of food availability, food composition, consumption patterns, and cultural factors. It has been mentioned that interventions of the health sector, particularly in Mexico, are a challenge to tackle obesity and overweight among the poor.^32,33^

In Mexico, it has been reported that adults have an inadequate intake of nutrients, among them are fats, carbohydrates, and micronutrients, such as A vitamin, C vitamin, and folic acid.^16,29^ Furthermore, it is suggested that other factors may influence this, such as socioeconomic status, and the greater proportion of subjects, both patients and controls showed a low socioeconomic level (Table 1). Also, we found a higher energy consumption of lipids and carbohydrates (Table 2). In a case–control study,^30^ an increase in carbohydrate intake was reported in patients with PD; the authors suggest that patients are likely to have developed a preference for certain foods and increased caloric intake of carbohydrates may represent a factor of importance for pathophysiological processes of the disease.^32–34^ In our study, we found no differences in carbohydrate intake between patients and controls, but the values are high. Therefore, it is likely that this condition in the control group, in addition to physical inactivity, might be seen reflected in being overweight. It has been reported that the concentrations of MUFA and PUFA could be related to the properties of E and C vitamins. This suggests that adequate levels of these vitamins may arise during the regeneration process of tocopherol, since C vitamin could enhance the antioxidant activity.^14^ In this paper, we evaluate the intake of fats and antioxidant vitamins in patients with neurodegeneration and these results support the preventive action of antioxidant vitamin intake. We found an increase in C vitamin consumption in the control group, related to a high intake of fruits and vegetables in this study group (Table 3), we suggested that this study could be related to the characteristics of population as this is a region where fruits and vegetables are easily obtained. This suggests a possible link with the demographic, geographical, and agricultural factors found in the region. The production of fruits and vegetables including, tomato, lime, lemon, guava, and alfalfa,^35^ which contain C vitamin was reported. C vitamin is a very potent antioxidant in plasma; however, their neuroprotective potential can be limited because it is hydrosoluble and requires an active transport in the choroid plexus to enter the CNS.^36^ It is reported that the average intake of vitamin C in rural Mexico is 60.7 mg/day.^16^ In our study, we found a higher intake (Table 2). This fact may be due to its geographical status and farm production in this region. Furthermore, considering the period when the survey was conducted, a questionnaire reported that subjects consumed a total of up to 15 servings of fruit in a high period of intake of fruits and vegetables, and oils and fats in the control group. These results are different from those reported in a study, which evaluates food consumption in the adult population.^37^ Rural people under conditions of insufficiency consume local farm products, and in seasonal production of fruits, they can consume high amounts.

This may be due to the lifestyle related to the behaviors of C vitamin intake rather than C vitamin itself, for example, consumption of sweet foods, including fruits containing C vitamin, may be associated with a high probability of risk to diseases such as neurodegeneration. In this study, we found a high intake of sugar in patients compared with the control group; patients reported a family history of chronic diseases in greater proportion, including diabetes mellitus, cardiovascular disease, and hypertension, which is consistent with that reported in the last census in the state of Jalisco.^20^ According to OMS, the BMI and energy intake are parameters used to evaluate nutritional status; in older adults daily macronutrient requirements are: 15% protein, 25% fat, and 60% carbohydrates.^26^ Patients included in this study reported a higher consumption of lipids (Table 2). This could be due to the fact that people in rural areas have a diet poor in necessary nutrients, so it is suggested to recommend foods rich in essential fatty acids such as fish and oleaginous. The dietary survey revealed that patients have a higher consumption of dairy products and cereals and have a lower intake of fruits and vegetables, which may indicate that they do not acquire sufficient minerals and vitamins from vegetables. Also, it has been suggested that a diet rich in high-glycemic foods is related to insulin resistance, a significant risk factor for diabetes mellitus and the development of obesity. In this study, the population presents in family history diabetes and cardiovascular disease; this is consistent with that reported in INEGI 2012,^38^ as it indicated that in Jalisco those are the main diseases in older adults. Chronic diseases such as diabetes mellitus, cardiovascular disease, obesity, and neurodegenerative diseases, such as AD and PD, are associated with diet and an unhealthy lifestyle.^39,40^ According to INEGI and ESANUT 2012, elderly people in Mexico present a higher percentage of being overweight, obesity, hypercholesterolemia, hypertension, and type 2 diabetes mellitus.^20,37^ In recent years, México reported an increase in the elderly population (9.2%). In 2050, it is estimated that this age group will make up about 28% of the population, which represents a major challenge for the health sector. In the state of Jalisco, this age group makes up 7.7–9.6% according to the ENSANUT, 2012.^41^ México presents a complex state of morbidity and mortality, in which non-communicable chronic diseases have become a major health problem in the adult population;^42,43^ within these changes and in relation to aging neurodegenerative diseases have become a major health problem.^41^

A deficiency of antioxidants such as C vitamins and E and beta-carotene, as well as nutrition-related disorders such as hypercholesterolemia, hypertension, and diabetes, may also play a role in cognitive impairment. These factors may be present for a long time before cognitive impairment becomes evident. Therefore, this could potentially be detected and corrected. The high value in saturated fat consumption found in adults relates to the food group of fats and oils (Table 3); among these are corn oil and safflower oil. While in the group of patients, we found a greater consumption of high-animal fat diet, in addition to, whole milk and cereal (Table 3), which indicates that this could be linked with developing a disease. ENSANUT 2006 reported an average consumption of the adult population of 40 g of fat per day in rural areas and a consumption of 10 g of MUFA and 6.9 g of PUFA.^29^ In this study, we found a higher consumption of lipids in the control group, slightly increased in comparison with the patients. We found a higher intake of C vitamin, A vitamin, and folic acid in the control group. However, these values are higher compared to the national average found in adults. 82.5 g of C vitamin, A vitamin 455.7 µg, and folic acid 234.5 µg,^16^ these deficiencies in antioxidant vitamins could be explained by the type of diet in these patients. However, it is important to intervene with nutrition education programs and actions that are committed to improving the quality of life for this age group.

The typical components of a Mexican diet include: carbohydrates contained in beans and corn; heavy consumption of meats, fats, oils, and sugar; and low consumption of fruits and vegetables.^35,43,44^ This high consumption of fat combined with a low consumption of fruits and vegetables could be contributing to the prevalence of obesity and related chronic diseases in the region. Fruit and vegetable intake play an important role in preventing overweight and obesity.^15,35^

This study of the patients reported an increase of cereals consumption, may be due to changes in the diet at the start of diagnosis in order to solve some alterations including decreased intestinal motility, which is a common symptom in patients with PD.^45^

The high consumption of whole milk observed in patients with neurodegeneration can be due to socioeconomic factors which already has resulted in cheaper rural areas access to whole milk to skim milk or skim. It has been reported that consumption of whole fat dairy products may be associated with cognitive impairment in older adults, so this finding would support this.^46^ It has also been reported that moderate consumption of saturated fat in young people has protective effects, while saturated fat intake may increase the risk of dementia and AD.^47^

In relation to alcohol consumption shown in patients, we proposed that this result may be due to the conditions where patients report that sometimes they consumed some alcoholic drinks especially when there are social gatherings. It has been reported that patients with PD will be inclined to reduce the consumption of alcohol at the time of diagnosis.^48^ Also, in case–control studies, there are limitations in the retrospective assessment of alcohol intake and selection bias. On the other hand, there are also studies that reported the intake of alcohol as a protective effect to PD; however, the results are still inconclusive.^49^ Intake of alcohol can contribute to oxidative stress, and its excess causes temporary or permanent cognitive impairment, and is associated with brain atrophy.^50^ Low intake of fruits and vegetables, observed in patients, could be related to the decrease in the protective effect (antioxidant) that these exert; thus, it could promote the development of neurodegeneration.^51^

In addition, a low intake of these foods has a detrimental effect on mineral and vitamin status, with negative consequences on health.^44,52^ However, their consumption has been inadequate in some countries, particularly in low and middle-class salary countries.^31^ In conclusion, this study indicates that older adults present a high intake of C vitamin; this is consistent with the food groups, such as fruits and vegetables for a seasonal period which could be related to the sociodemographic characteristics of this region. The patients with possible AD or PD had a lower intake of fruits and vegetables, which could be due to the type of food to which they have access.
